# SIRT1 reduces endothelial activation without affecting vascular function in ApoE-/- mice

**DOI:** 10.18632/aging.100162

**Published:** 2010-06-28

**Authors:** Sokrates Stein, Nicola Schäfer, Alexander Breitenstein, Christian Besler, Stephan Winnik, Christine Lohmann, Kathrin Heinrich, Chad E. Brokopp, Christoph Handschin, Ulf Landmesser, Felix C. Tanner, Thomas F. Lüscher, Christian M. Matter

**Affiliations:** ^1^ Cardiovascular Research, Institute of Physiology, Zurich Center for Integrative Human Physiology (ZIHP), University of Zurich, and Cardiovascular Center, Cardiology, University Hospital Zurich, CH-8057 Zurich, Switzerland; ^2^ Biozentrum, University of Basel, CH-4056 Basel, Switzerland

**Keywords:** SIRT1, atherosclerosis, endothelium, inflammation

## Abstract

Excessive production of reactive oxygen species (ROS) contributes to progression of atherosclerosis, at least in part by causing endothelial dysfunction and inflammatory activation. The class III histone deacetylase SIRT1 has been implicated in extension of lifespan. In the vasculature,SIRT1 gain-of-function using SIRT1 overexpression or activation has been shown to improve endothelial function in mice and rats via stimulation of endothelial nitric oxide (NO) synthase (eNOS). However, the effects of SIRT1 loss-of-function on the endothelium in atherosclerosis remain to be characterized. Thus, we have investigated the endothelial effects of decreased endogenous SIRT1 in hypercholesterolemic ApoE^-/-^ mice. We observed no difference in endothelial relaxation and eNOS (Ser^1177^) phosphorylation between 20-week old male atherosclerotic ApoE^-/-^ SIRT1^+/-^ and ApoE^-/-^ SIRT1^+/+^ mice. However, SIRT1 prevented endothelial superoxide production, inhibited NF-κB signaling, and diminished expression of adhesion molecules. Treatment of young hypercholesterolemic ApoE^-/-^ SIRT1^+/-^ mice with lipopolysaccharide to boost NF-κB signaling led to a more pronounced endothelial expression of ICAM-1 and VCAM-1 as compared to ApoE^-/-^ SIRT1^+/+^ mice. In conclusion, endogenous SIRT1 diminishes endothelial activation in ApoE^-/-^ mice, but does not affect endothelium-dependent vasodilatation.

## Introduction

Inflammation plays a key role in the development and progression of atherosclerosis. In early stages of the disease, endothelial cells get activated by circulating proinflammatory molecules such as cytokines (e.g. TNFα) or modified lipoproteins (e.g. oxidized LDL). Once activated, these cells express chemokines, cytokines, and adhesion molecules, which attract and recruit inflammatory cells such as macrophages and T cells [1,2]. Hypertension, hypercholesterolemia, diabetes, and aging, which may all be associated with an excessive production of reactive oxygen species (ROS) and oxidant stress, may contribute to atherosclerosis by affecting endothelial function and inducing sustained endothelial activation [2-5].

The NAD-dependent class III histone deacetylase Sir2 was found to increase lifespan in yeast [6]. Its mammalian orthologue SIRT1 senses caloric restriction, improves insulin secretion in pancreatic beta cells, and reduces accumulation of fatty acids in white adipose tissue [7-9]. Various other SIRT1 targets have been identified and characterized in recent years, including PGC-1α, NF-κB and LXR [10-13]. NF-κB is of special interest in endothelial cells, since it drives the expression of important adhesion molecules, such as vascular cell adhesion molecule-1 (VCAM-1) and intercellular adhesion molecule-1 (ICAM-1), which recruit blood monocytes to atherosclerotic lesions [14-16].

Endogenous SIRT1 has been shown to decrease macrophage foam cell formation and atherogenesis in hypercholesterolemic *ApoE^-/-^ SIRT1^+/-^* mice [17]. In non-atherosclerotic aortae of rats, dominant-negative SIRT1 transfection impairs endothelial function via eNOS inhibition *ex vivo*[18], and endothelial overexpression of human SIRT1 diminishes atherogenesis in *ApoE^-/-^* mice and improves vascular function [19]. In addition, activation of SIRT1 prevents hyperglycemia-induced vascular cell senescence in mice with diabetes, thereby protecting from vascular dysfunction [20]. Nevertheless, the impact of a *SIRT1 *haploinsufficiency on endothelium-dependent vaso-motion and endothelial cell activation in atherosclerotic mice remains to be determined.

In the present study, we therefore investigated the effects of a single SIRT1 allele on aortic relaxation and endothelial activation in 20-week-old atherosclerotic *ApoE^-/-^ SIRT1^+/+^* and *ApoE^-/-^ SIRT1^+/-^* mice.

**Figure 1.**
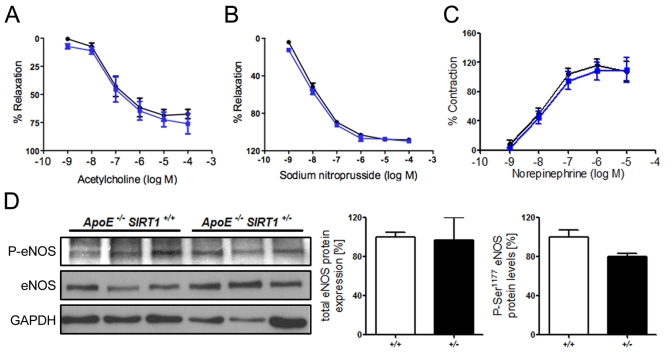
(**A**) No difference in relaxation of aortic rings preconstricted with norepinephrine to the vasodilator acetylcholine. % Relaxation = % of precontraction to norepinephrine. (**B**) Relaxation of aortic rings at increasing sodium nitroprusside concentrations after norepinephrine precontraction. % Relaxation = % of precontraction to norepinephrine. (**C**) Contraction of aortic rings at increasing norepinephrine concentrations. % Contraction = % of contraction to 80 mM KCl. *ApoE^-/-^ SIRT1^+/-^* (blue line) and *ApoE^-/-^ SIRT1^+/+^* (black line). (**D**) Aortic protein levels of total eNOS and phospho-eNOS (Ser1177). *ApoE^-/-^ SIRT1^+/-^* (+/- and black columns) and *ApoE^-/-^ SIRT1^+/+^* (+/+ and white columns). n=6 per genotype

## Results

Endogenous SIRT1 does not alter endothelial function in *ApoE^-/-^* mice Overexpression of human SIRT1 in mouse endothelial cells has been shown to diminish atherogenesis in *ApoE^-/-^* mice. [19] However, the underlying mechanisms remain to be further characterized. To investigate the effect of endogenous *SIRT1* on endothelium-dependent vasodilatation and endothelial inflammatory activation, we assessed endothelium-dependent function and inflammatory pathways in aortic rings from 20-week-old atherosclerotic *ApoE^-/-^ SIRT1^+/+^* or *ApoE^-/-^ SIRT1^+/-^* mice. Interestingly, the acetylcholine-mediated relaxation of aortic rings after precontraction with norepinephrine did not differ between *ApoE^-/-^ SIRT1^+/+^* and the haploinsufficient *ApoE^-/-^ SIRT1^+/-^* mice (Figure 1A). Vasoconstriction with norepinephrine and endothelium-independent vasodilatation with sodium nitroprusside were normal (Figure 1B, C). eNOS-derived NO plays an important role in vascular relaxation, and eNOS activity is mainly regulated by Akt-dependent Ser^1177^ phosphorylation [21]. We observed no difference in the Ser^1177^ phosphorylation status (Figure 1D). Our data indicate that endogenous SIRT1 in atherosclerotic *ApoE^-/-^* mice does not affect endothelial function.

**Figure 2.**
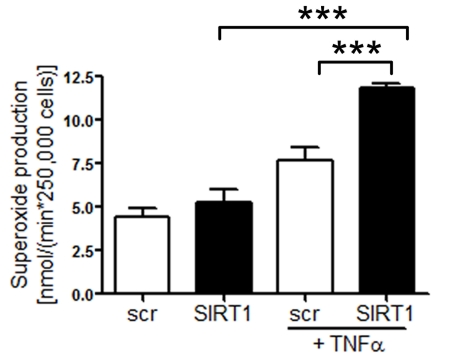
Superoxide production is increased in HAECs after SIRT1-siRNA compared with scrambled-siRNA-treatment 1 h after TNFα stimulation. n=2. ***p<0.001.

Silencing of SIRT1 enhances production of endothelial superoxide Common risk factors predisposing to atherosclerosis, such as hypercholesterolemia or aging, are associated with oxidant stress at least in part due to an increased production of ROS [22]. We measured ROS production in human aortic endothelial cells (HAECs) treated with either scrambled- or SIRT1-siRNA. SIRT1 silencing elevated endothelial ROS levels upon TNFα stimulation, whereas under basal conditions there was no effect of SIRT1 silencing was observed (Figure 2).

**Figure 3.**
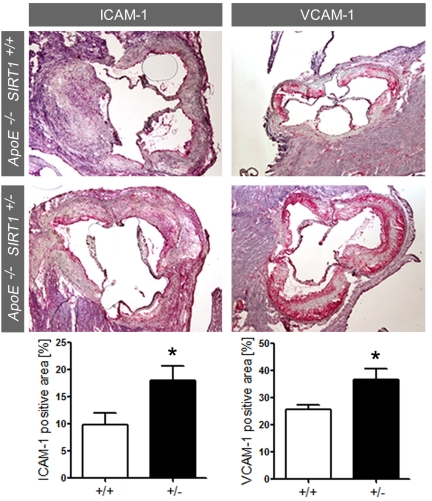
ICAM-1 and VCAM-1 staining and quantification in plaques from aortic sinus. Magnifications: X40. *ApoE^-/-^ SIRT1^+/+^* (+/+, n=6, white columns) and *ApoE^-/-^ SIRT1^+/-^* (+/-, n=6, black columns). *p<0.05.

Enhanced expression of adhesion molecules in *ApoE^-/-^ SIRT1^+/-^* plaques Accumulating evidence suggests that chronic production of ROS may favor atherogenesis by inducing sustained endothelial inflammatory activation [2,5]. Expression of endothelial adhesion molecules play an important role in atherogenesis by promoting monocyte-derived macrophage recruitment and accumulation in the arterial intima [16]. Interestingly, expression of ICAM-1 and VCAM-1 was increased in atherosclerotic plaques of *ApoE^-/-^ SIRT1^+/-^* compared with *ApoE^-/-^ SIRT1^+/+^*mice (Figure 3). These findings show that SIRT1 prevents adhesion molecule expression, an important step in endothelial cell activation.

**Figure 4.**
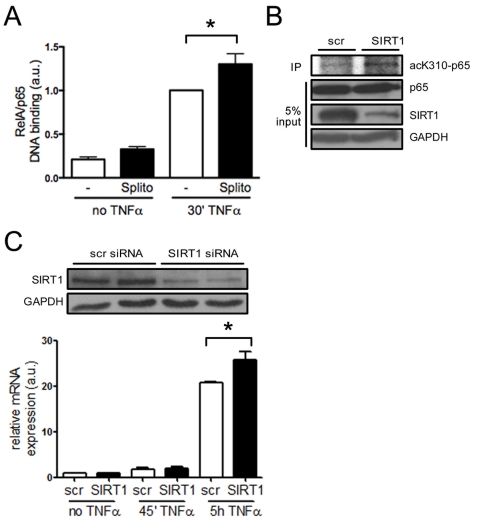
SIRT1 suppresses NF-κB signaling in HAECs. (**A**) RelA/p65 DNA binding is higher in HAEC pretreated with splitomicin (Splito) compared with the untreated group (-), 30 min after TNFα-stimulation. n=5. (**B**) RelA/p65 immunoprecipitation in HAECs reveals more acetyl-K310-RelA/p65 upon SIRT1-siRNA treatment 20 min after TNFα-stimulation compared to scrambled siRNA-treated cells. n=2. (**C**) Western blot showing SIRT1 silencing using siRNA (top), and VCAM-1 mRNA expression 5 h after TNFα stimulation in SIRT1-siRNA treated HAECs (graph). n=4. *p<0.05.

SIRT1 regulates the expression of endothelial adhesion molecules via suppression of NF-κB signaling *in vitro *NF-κB plays a central role in inflammatory processes and its signaling pathway is inhibited by SIRT1 via deacetylation [12,23]. NF-κB induces expression of adhesion molecules and inflammatory cytokines, and endothelial-specific inhibition of the NF-κB pathway protects mice from atherosclerosis [24]. SIRT1 has been shown to deacetylate the lysine residue K310 of RelA/p65 in human epithelial lung cells [12]. To test whether RelA/p65 signaling is suppressed by SIRT1 in HAECs, we quantified DNA-bound RelA/p65 in TNFα-stimulated and unstimulated cells pretreated with the SIRT1 inhibitor splitomicin [25]. Binding of RelA/p65 to naked DNA was enhanced upon treatment with the SIRT1 inhibitor splitomicin after TNFα stimulation (Figure 4A). To evaluate, if SIRT1 is also deacetylating K310 of RelA/p65 in HAECs, as previously reported for HEK 293T cells [12], we stimulated SIRT1- or scrambled-siRNA-treated HAECs with TNFα and performed p65 immunoprecipitations. K310-p65 was increased in SIRT1-siRNA-treated HAECs (Figure 4B). To further test if suppression of NF-κB signaling also affects the expression of adhesion molecules, we analyzed the expression of VCAM-1, a known NF-κB signaling target, in more detail. SIRT1-siRNA treatment enhanced expression of VCAM-1 in HAECs upon TNFα stimulation (Figure 4C).

**Figure 5.**
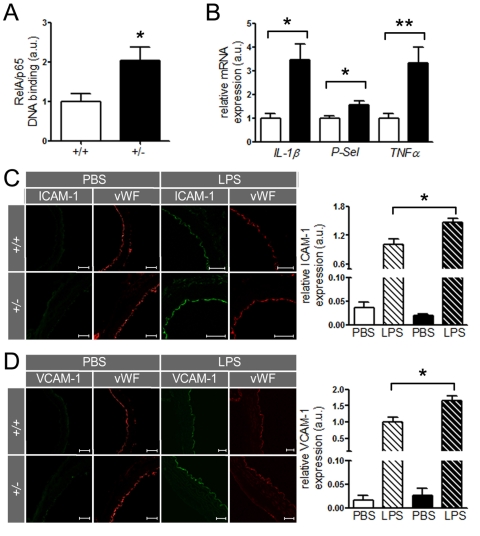
SIRT1 prevents expression of endothelial adhesion molecules. (**A**) RelA/p65 DNA-binding in aortic nuclear extracts from *ApoE^-/-^ SIRT1^+/- ^*(+/-, n=8, black column) is elevated in *ApoE^-/-^ SIRT1^+/+^* (+/+, n=6, white column) mice. (**B**) Expression levels of *IL-1β*, *P-Selectin*, and *TNFα* in aortic lysates of *ApoE^-/-^ SIRT1^+/+^* (white columns) and *ApoE^-/-^ SIRT1^+/-^* (black columns) mice. n=8 per genotype. Enhanced expression of ICAM-1 (**C**) and VCAM-1 (**D**) is observed in non-atherosclerotic *ApoE^-/-^ SIRT1^+/-^* (+/- and black columns) compared with *ApoE^-/-^ SIRT1^+/+^* (+/+ and white columns) aortae 3 h post intra-peritoneal LPS (striped columns) injection. n=6 per genotype. Scale: 50 μm. *p<0.05; **p< 0.01.

SIRT1 regulates the expression of inflammatory endothelial molecules *in vivo *Since SIRT1 suppresses NF-κB signaling in HAECs, we investigated whether the same concept holds true in mouse aortae. Binding of RelA/p65 to naked DNA was higher in nuclear extracts of *ApoE^-/-^ SIRT1^+/- ^*than* ApoE^-/-^ SIRT1^+/+^* mice (Figure 5A). Importantly, aortic expression of other inflammatory molecules, namely *IL-1β*,*TNFα*, and *P-Selectin* (*P-Sel*), was also enhanced in *ApoE^-/-^ SIRT1^+/- ^*compared to* ApoE^-/-^ SIRT1^+/+^* mice (Figure 5B). The expression of these inflammatory genes is regulated by NF-κB. Lipopolysaccharides (LPS) induce strong activation of NF-κB signaling and the expression of target genes [26]. To address the *in vivo* relevance of the NF-κB suppression by SIRT1, we examined the expression of two known NF-κB-dependent genes, ICAM-1 and VCAM-1, in aortae from young 8-week-old *ApoE^-/-^ SIRT1^+/-^* and *ApoE^-/-^ SIRT1^+/+^* mice without atherosclerosis in descending thoracic aortae 3 hours after intraperitoneal injection of LPS. LPS induced an upregulation of both ICAM-1 and VCAM-1 in intimal endothelial cells of aortae from *ApoE^-/-^ SIRT1^+/-^* compared with *ApoE^-/-^ SIRT1^+/+^* mice (Figure 5C, D). These findings indicate that endogenous SIRT1 is sufficient to prevent adhesion molecule expression in both human and mouse activated endothelial cells.

## Discussion

Enhanced atherogenesis in *ApoE^-/-^ SIRT1^+/-^* mice is causally linked to increased expression of adhesion molecules in aortae. Indeed, we provide *in vitro* and *in vivo* evidence that underlines this concept by demonstrating that *ApoE^-/-^ SIRT1^+/-^* mice exhibit increased endothelial expression of ICAM-1 and VCAM-1 upon LPS injection. Importantly, upregulation of these adhesion molecules promotes recruitment of monocytes and T cells to luminal endothelial cells [27]. In concert with increased levels of IL-1β, TNFα, and P-Sel in the activated arterial wall, these molecular events are sufficient to recruit circulating leukocytes to atherosclerotic lesions, especially monocyte-derived macrophages and T cells.

At the molecular level, the inhibitory effects of SIRT1 on adhesion molecule expression may be mediated via RelA/p65 signaling**.**Our data show that SIRT1 suppresses binding of RelA/p65 to naked DNA, therefore interfering with a crucial step in the transcriptional activation of NF-κB. These findings are in line with previous reports showing that SIRT1 deacetylases RelA/p65 at the lysine residue K310 in human epithelial lung cells [12]. In agreement with these reports, we demonstrate that this mechanism of RelA/p65 signaling suppression is present in HAECs.

Surprisingly, we observe no endothelial dysfunction in *ApoE^-/-^ SIRT1^+/-^* mice. In contrast, Pearson et al. showed improved endothelial function in mice kept on a diet with a very high resveratrol content (2400 mg/kg/food) that could be mediated by SIRT1 activation [28]. However, such effects may also be related to activation of AMPK by resveratrol or via other targets of this compound [29,30]. Furthermore, adenovirus-mediated inhibition of endothelial SIRT1 diminishes endothelium-dependent vasodilatation in rat aortic rings and decreases bioavailable NO levels [18]. Others reported improved relaxation in *ApoE^-/-^* mice with endothelial*SIRT1* overexpression that were kept on a high-fat diet [19]. However, in this study WT aortic rings showed also marked endothelial dysfunction by relaxing only up to 50%, thereby casting doubts on the endothelial integrity of the preparations [19]. In contrast, we observed no change in endothelial function or aortic eNOS activity between hyper-cholesterolemic *ApoE^-/-^ SIRT1^+/-^* and *ApoE^-/-^ SIRT1^+/+ ^*mice, suggesting that the endothelial-protective effects of SIRT1 include factors other than eNOS-dependent NO production. Indeed, we detected a profound increase in ROS-production after silencing of SIRT1 in TNFalpha-stimulated endothelial cells, indicating that endogenous SIRT1 inhibits agonist-induced ROS production in endothelial cells. Of note, an excessive production of ROS has been implicated in endothelial inflammatory activation and the pathogenesis of atherosclerosis [31]. Therefore, inhibition of excessive endothelial ROS production likely represents an important endothelial-protective action of endogenous SIRT1.

Taken together, our results show that SIRT1 does not influence endothelium-dependent vascular function in *ApoE^-/-^* mice, but it prevents superoxide production in endothelial cells and reduces the expression of inflammatory adhesion molecules by suppressing NF-κB signaling. Although the specificity of available SIRT1 activators has been questioned recently [32], it is likely that SIRT1 activators may prevent atherosclerosis and other inflammatory diseases by hindering pro-oxidative and inflammatory processes.

## Materials and methods

Animals. *ApoE^-/-^ SIRT1^+/-^* and *ApoE^-/-^ SIRT1^+/+^* mice were described previously [17]. Male mice were fed a high-cholesterol diet (1.25% total cholesterol, Research Diets) for 12 weeks starting at the age of 8 weeks. All animal procedures were approved by the local animal committee and performed in accordance with our institutional guidelines.

Cell culture. Human aortic endothelial cells (HAEC, Cambrex Bio Science) were treated with 100 μM splitomicin (Sigma-Aldrich) to perform analysis of NF-κB binding to DNA. HAEC were stimulated for 30 minutes with 10 ng/ml human TNFα (R&D Systems).

siRNA transfection. Transient transfection siRNA into HAEC were done with lipofectamin lipofectamin RNAi MAX (Invitrogen). The oligos used for SIRT1-siRNA have been described previously [9].

Immunohistochemistry and immunofluorescence. Serial cryosections from the aortic sinus were stained with rabbit anti-von Willebrand Factor (Dako), rat anti-CD31, rat anti-VCAM-1 (BD Biosciences), rat anti-ICAM-1 (Serotec). Fluorescence was analyzed on a Leica TCS SP2 confocal microscope and means were taken from n=6 different mice evaluating 6 serial cryosections/tissue from each mouse.

RNA and protein analysis. Total RNA isolated from proximal aortae and HAEC was extracted with TRIZOL (Invitrogen), reverse transcribed, and the cDNA quantified by SYBR green qPCR using specific primers. For protein analysis, aortic tissue lysates were blotted and incubated with rabbit anti-SIRT1, rabbit anti-eNOS (Santa Cruz Biotechnology), and rabbit anti-Phospho-eNOS (Ser^1177^) (Cell Signaling Technology).

Endothelial function. Aortic rings (2-3 mm long) were connected to an isometric force transducer (MultiMyograph), suspended in a 95% O_2_/5% CO_2 _aerated organ chamber filled with KREBS buffer (118 mM NaCl, 4.7 mM KCl, 1.2 mM MgCl_2_, 1.2 mM NaH_2_PO_4_, 1.2 mM Na_2_SO_4_, 2.5 mM CaCl_2_, 25 mM NaHCO_3_, 10 mM glucose, pH to 7.4). Concentration-dependent contractions were established by using norepinephrine (10^−9^ to 10^−4^ mol/liter; Sigma-Aldrich).  Concentration-response curves were obtained in a cumulative fashion. 8 rings cut from the same artery were studied in parallel. Responses to acetylcholine (10^−9^ to 10^−6^ mol/liter; Sigma-Aldrich) were obtained during submaximal contraction to norepinephrine. The NO donor sodium nitroprusside (10^−10^ to 10^−5^ mol/liter; Sigma-Aldrich) was added to test endothelium-independent relaxations.

LPS assay. 6 8-week-old *ApoE^-/-^ SIRT1^+/-^* and *ApoE^-/-^ SIRT1^+/+^* mice kept on standard diet were used for this assay. At this age and under normal diet, *ApoE^-/-^* mice do not exhibit plaques in their thoraco-abdominal aortae. Mice were injected i.p. with 100 μg of LPS (Sigma) in PBS, sacrificed 3 hours post injection and thoraco-abdominal aortae embedded in OCT. Cryosections (5 μm) were cut and stained for ICAM-1 or VCAM-1. Relative expression is given as the ratio of ICAM-1 or VCAM-1 staining area to von Willebrand Factor (vWF) staining area, respectively. Quantification of fluorescence was done with analySIS (Olympus) on microscopic images using identical exposure settings.

Quantification of DNA-bound RelA/p65. HAEC were pretreated with splitomicin for one hour and stimulated with 10 ng/ml TNFα for 30 minutes. Nuclear extracts of aortic tissue samples were obtained with the Nuclear Extract kit (ActiveMotif) using a Dounce pestle, and a RelA/p65 transcription factor assay was performed using the TransAM kit (ActiveMotif) according to the manufacturer's instructions.

Immunoprecipitation. HAEC were treated with 50 μM SIRT1 or scrambled siRNA over night, followed by 10 ng/ml TNFα stimulation for 20 minutes. Cells were then harvested and protein extracted in lysis buffer (20 mM HEPES, pH 7.5, 80 mM NaCl, 2.5 mM MgCl2, 1 mM EDTA, 100 μM Splitomicin, 0.5% NP-40, 1 mM PMFS (phenylmethylsulfonyl fluoride), 10 μg/ml aprotinin, and 10 μg/ml leupeptin). 1 mg whole-cell lysates were immunoprecipitated with rabbit anti-p65 (Santa Cruz) using Protein G agarose (Millipore). Immunoprecipitated samples were immunoblotted with rabbit anti-acK310-p65 (Abcam), and the total lysates (5% input) with rabbit anti-SIRT1 and rabbit anti-p65.

Detection of endothelial cell superoxide production by electron spin resonance spectroscopy. The effect of SIRT1 on endothelial cell superoxide production was assessed in unstimulated and TNFα-stimulated (10 ng/ml) HAEC by ESR spectroscopy using the spin probe 1-hydroxy-3-methoxycarbonyl-2,2,5,5-tetra-methylpyrrolidine (CMH; Noxygen). HAEC were incubated with 50 nM scrambled or SIRT1-siRNA with or without TNFα for one hour and resuspended in Krebs-Hepes buffer (pH 7.4; Noxygen) containing diethyldithiocarbamic acid sodium salt (5 μM, Noxygen) and deferoxamine methanesulfonate salt (25 μM, Noxygen). ESR spectra were recorded after addition of CMH (final concentration 200 μM) under stable temperature conditions using a Bruker e-scan spectrometer (Bruker Biospin). The ESR instrumental settings were as follows: center field, 3495 G; field sweep width, 10.000 G; microwave frequency, 9.75 GHz; microwave power, 19.91 mW; magnetic field modulation frequency, 86.00 kHz; modulation amplitude, 2.60 G; conversion time, 10.24 msec; detector time constant, 328 msec; nuber of x-scans, 10.

Statistical analyses. Data are presented as mean ± SEM. Statistical significance of differences was calculated using an ANOVA with post hoc Tukey's test or Student's unpaired t test. Significance was accepted at p<0.05.

## Sources and funding

This work was funded by grants from the Swiss National Science Foundation (#31-114094/1, #310030_130626/1, and #3100-068118) and the University Research Priority Program "Integrative Human Physiology" at the University of Zurich.
